# Learning Word Meanings: Overnight Integration and Study Modality Effects

**DOI:** 10.1371/journal.pone.0124926

**Published:** 2015-05-19

**Authors:** Frauke van der Ven, Atsuko Takashima, Eliane Segers, Ludo Verhoeven

**Affiliations:** Behavioural Science Institute, Radboud University Nijmegen, Nijmegen, The Netherlands; University of Barcelona, SPAIN

## Abstract

According to the complementary learning systems (CLS) account of word learning, novel words are rapidly acquired (learning system 1), but slowly integrated into the mental lexicon (learning system 2). This two-step learning process has been shown to apply to novel word *forms*. In this study, we investigated whether novel word *meanings* are also gradually integrated after acquisition by measuring the extent to which newly learned words were able to prime semantically related words at two different time points. In addition, we investigated whether modality at study modulates this integration process. Sixty-four adult participants studied novel words together with written or spoken definitions. These words did not prime semantically related words directly following study, but did so after a 24-hour delay. This significant increase in the magnitude of the priming effect suggests that semantic integration occurs over time. Overall, words that were studied with a written definition showed larger priming effects, suggesting greater integration for the written study modality. Although the process of integration, reflected as an increase in the priming effect over time, did not significantly differ between study modalities, words studied with a written definition showed the most prominent positive effect after a 24-hour delay. Our data suggest that semantic integration requires time, and that studying in written format benefits semantic integration more than studying in spoken format. These findings are discussed in light of the CLS theory of word learning.

## Introduction

Imagine reading a story and encountering the word *sambar*. This word is unknown to you, but the dictionary states that a *sambar* is a “deer living in the forests of Southeast Asia”. Or, you ask a friend who gives you this definition. Adults continuously learn novel words, and these words become integrated in the network of words that a person already knows. Word definitions can be encountered in written (e.g., a dictionary definition) or spoken format (e.g., a friend’s definition). It is, however, by no means clear to what extent study modality has an impact on how people learn new word meanings. In the present study, we therefore investigated how novel word meanings are incorporated into the semantic lexicon, and how this integration process is influenced by the modality in which word meanings are learned.

### Integration of Novel Words Into the Mental Lexicon

Following dual-systems accounts in memory research (e.g., [1]), Davis and Gaskell [2] suggested that adult word learning occurs via two *complementary learning systems (CLS)*. The first learning system rapidly *acquires* a novel word and stores it as an episodic memory trace, independent of the already existing network of word representations. This memory system is supported by the medial temporal lobe, including the hippocampus. The second learning system encompasses several post-acquisition learning processes (also known as consolidation processes [3, 4]), such as stabilization (strengthening of a memory trace, e.g., [5]), generalization (extraction of gist/rules, e.g., [6]), and *integration* (formation of new relations between novel and old knowledge). For post-acquisition learning processes, such as integration, sleep is thought to be important [2–4, 7]. Although the CLS account of word learning was developed with the aim to cover a broad spectrum of word-learning phenomena, it is mainly founded on evidence from paradigms involving the learning of word *forms*. That is, a novel word, such as *sambar*, is swiftly acquired, but only starts to influence the processing of perceptually similar word forms, such as *samba*, after a consolidation period. This delay has been interpreted to reflect slow integration into the mental lexicon. However, it remains unclear whether the CLS theory also applies to the learning of word *meanings*. To establish whether integration of word meanings into the semantic lexicon involves a similar post-acquisition process, we examined whether a novel word such as *sambar* influences the processing of semantically related words such as *antlers* over time.

A *primed lexical decision task (pLDT)* can reveal the interaction of words that are connected within the semantic network (see [8] for a review on semantic priming). In a pLDT, one has to decide whether a string of letters (the target) is an existing word or not (i.e., the lexical decision). Responses are typically faster when an existing target (e.g., *antlers*) is preceded by a semantically related prime (e.g., *moose*) than when it is preceded by an unrelated prime (e.g., *chair*). This reduction in reaction time (RT) is known as the *priming effect*. The speeded lexical decision is thought to be caused by activation spreading from the prime to semantically related words in the mental lexicon, including the related target word [9]. The priming effect can be employed for investigating word learning, because it can be used as a measurement of *semantic integration*: if a newly learned word (e.g., *sambar*) is able to prime a word that is related in meaning (e.g., *antlers*), this is evidence for the word being semantically integrated.

Semantic priming is usually observed when prime and target words have closely overlapping meanings (i.e., *semantic relation*, e.g., mouse-rat). This effect can also be observed if words share only few semantic features, but are repetitively experienced together (i.e., *semantic association*, e.g., mouse-cheese). Priming has even been shown for word pairs that were pre-experimentally unrelated (e.g., mouse-puzzle), but co-occurred in a study list just prior to the priming task [10]. The latter suggests that, if prime-target connections have just been experienced, priming can result from the episodic (not yet integrated) memory of prime-target connections, rather than that priming reflects connections in the semantic lexicon (see [11], for a similar argument). In other words, if novel words—the primes—are studied in combination with their targets prior to a priming experiment (e.g., [12, 13]), an observed priming effect could reflect an intact episodic memory trace rather than the integration of novel words into the semantic memory system. Hence, in order to use the priming effect as a measure of semantic integration, the possibility of episodic coupling of prime and target should be minimized.

Two investigations of semantic integration with a design that avoided the occurrence of episodic coupling during study have been reported by Tamminen and Gaskell [11] and by Clay, Bowers, Davis, and Hanley [14]. Tamminen and Gaskell, whose study consisted of two semantic priming experiments, found that newly learned words prime semantically related words even if the prime-target word pairings do not occur during study. This finding evidences integration of novel words with existing words in the semantic lexicon. A combined analysis of the two priming experiments showed more integration over time. Clay et al. used a picture-word interference task (PWIT) to investigate semantic integration. The PWIT is a Stroop-like task in which a picture is presented together with a word. Words that are semantically related to the picture usually slow down the picture naming reaction times compared to words that are not semantically related. Directly after study, newly learned words did not show a semantic interference effect, but this effect was observed after approximately one week, indicating that consolidation plays a role in word integration. In short, these two studies suggest that semantic integration occurs over time, via a consolidation process that follows initial acquisition of the novel words.

Although many of the potential confounds were avoided in the two studies mentioned above, in both designs the newly learned words that were allowed the longest consolidation period may have also been studied more “intensively”, because of spaced or repeated study options. In Tamminen and Gaskell’s study [11], experiment 1, a recall task that was applied before the pLDT, may have led to spaced learning. In experiment 2, this issue was addressed, and the authors concluded no effect of the recall task on priming. However in experiment 2 (not in 1), even though the primes were masked, repetition of prime-target pairs across the pLDT sessions may have led to both spaced and repeated learning. This means that the authors did not observe an effect of the recall test on priming *on top* of the possible effects that the repeated pLDTs might have had. In a similar vein, the words in the long-consolidation condition in the study of Clay et al. [14] may have had more opportunities for intensive study, because the picture-word pairs were repeated in the PWITs. This makes it hard to discern whether the interference effects found in the delayed PWIT were due to post-acquisition processes or to extra practice of the novel words provided by the PWIT that was applied directly after study.

In sum, the CLS account of word learning supposes that a word is rapidly acquired, but integrated via a slower consolidation process. There is not much evidence yet to support the existence of these two learning systems in the learning of word meanings, although the available data are in line with this view [11, 14]. With this study, we aimed to provide evidence for the two-step learning of novel meaningful words, while carefully controlling for the amount and style of encoding.

### Study Modality Effects on Word Learning

Definitions of words can be encountered in written or spoken format. If a specific study modality causes novel information to become more easily incorporated into the student’s existing knowledge, this has direct educational implications since teachers can choose to provide either written or spoken explanations of novel concepts. Literature on whether study modality has an influence on adult novel word learning is scarce.

Past studies on memory for familiar words show that short-term memory benefits from a spoken study format, because of a larger recency effect that is possibly due to a larger capacity for verbal working memory than for visual working memory [15]. When slightly longer retention intervals are used, results become very diverse, with some researchers reporting a memory advantage for spoken words [16] and others for written words [17]. Evidence thus suggests a memory advantage for spoken material on the very short term, which might disappear when the interval between study and test is prolonged. Study modality effects on novel word learning, however, are relatively understudied.

Regarding modality effects on novel word learning, it appears that written study material leads to better word-learning performance in adults than spoken material, causing stronger representations of word form and stronger couplings with word meaning [18, 19]. If longer time intervals are used between study and test, this advantage may disappear if learners are proficient in the to-be-learned language [20]. There is only little information about study modality effects on adult first language acquisition. Moreover, we are not aware of studies to date investigating the influence of study modality on the *integration* of novel meaningful words.

### The Present Study

The CLS account of word learning suggests that words are rapidly acquired and slowly integrated [2], but the evidence for the application of this model to the learning of word meanings is limited. Furthermore, it is unknown how study modality affects the integration of novel words into the semantic lexicon. With the present study, we aimed to establish 1) whether newly learned (novel) words require time to become semantically integrated, and 2) to what extent this integration process is influenced by study modality. In order to investigate the integration trajectory of newly learned words, we used the semantic priming effect to reflect the degree of semantic integration. Participants studied visually presented novel and known words with their meanings (definitions). These studied words served as primes for semantically related and unrelated targets in two pLDTs. The first pLDT was applied immediately after study, and the second pLDT after a 24-hour delay. Our hypothesis was that novel words become slowly integrated into the semantic lexicon, and should therefore serve as stronger primes after a consolidation period. To probe our second question, the definitions were presented in written format for one group and in spoken format for another group of participants. We hypothesized that if the integration process is influenced by study modality, the written modality group should outperform the spoken modality group, resulting in a stronger priming effect for the written modality on Day 2. Known study words, which were expected to already be part of the semantic lexicon, were included as a control condition to confirm if our task was sensitive enough to show priming effects and to check for possible differences in baseline priming for the different days and modality groups.

## Materials and Methods

### Participants

Sixty-four native Dutch-speakers were recruited from the university campus and surroundings. Sixty-three participants were students at an (applied) university or had finished this education. One participant finished secondary education. His performance was not exceptionally different from others. None of the participants reported to be visually impaired or to have reading or hearing problems. Ethical approval was obtained from the Ethics Committee Faculty of Social Sciences of the Radboud University Nijmegen. All participants gave written consent and received study credits or money for participation. Half of the participants were assigned to the written modality group and the other half to the spoken modality group (written: *n* = 32, 24 females, 26 right handed, *M*_age_ = 24.64 years, age range = 18–64 years; spoken: *n* = 32, 30 females, 27 right handed, *M*_age_ = 22.53 years, age range = 18–36 years).

### Procedure

**Fig 1:**
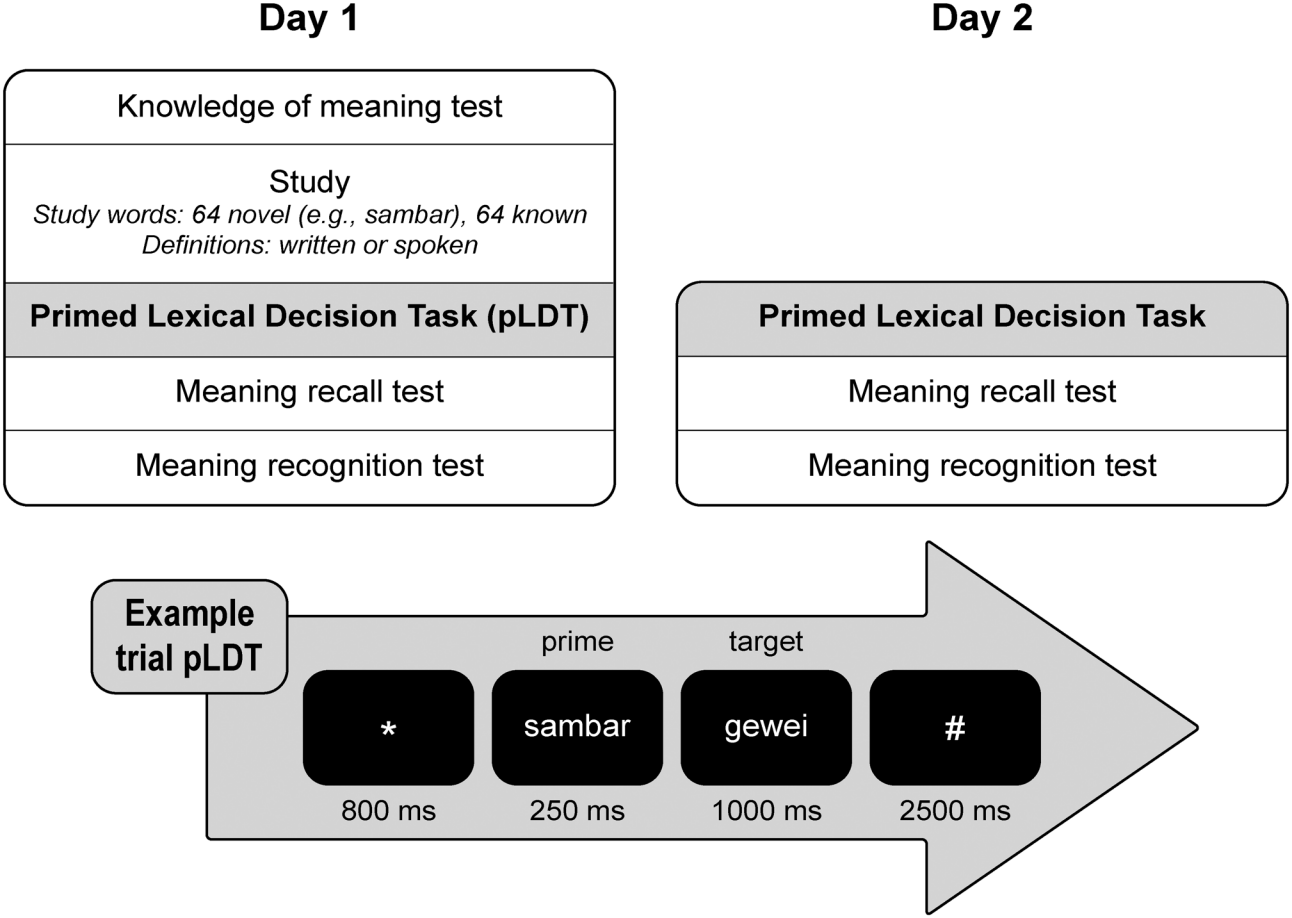
Procedure. On Day 1 participants were tested on the knowledge of the meaning of the words they were about to study. In the study phase, 64 novel and 64 known written words were studied, with their definitions (meaning) presented in written or spoken format (Modality = between-participants factor). Half of the studied words served as primes in a primed lexical decision task (pLDT) on Day 1 and the other half on Day 2 (Day = within-participants factor). Both pLDTs were followed by a meaning recall and meaning recognition test of the words that had just been used as primes in the pLDT. An example trial of a related prime-target pair in the pLDT is shown in the lower part of the figure; participants had to indicate whether a target was an existing Dutch word or not (lexical decision), and the target (e.g., *gewei* = antlers in Dutch) was primed with one of the studied words (e.g., *sambar*).

On Day 1, participants were first tested on their knowledge of both the novel and known study words (Fig 1). Next, they studied novel and known written words with their definitions. Definitions were presented in written format for the written modality group and in spoken format for the spoken modality group. To investigate integration of novel words into the semantic lexicon, participants took part in a pLDT on both Day 1 (+/- 10 minutes after study) and Day 2 (approximately 24 hours after study). Testing occurred during daytime (8 AM–6 PM), but within participants the time of testing was kept constant (*M*_time difference pLDT Day 1 and Day 2_ (*SD*): written condition: 24 hr 8 min (30 min); spoken: 23 hr 59 min (21 min)). Half of the studied words served as primes for target words in the pLDT on Day 1, and the other half in the pLDT on Day 2, thus avoiding the repetition of prime-target occurrence across the two test points. Directly after these pLDTs, participants were tested on recall (subjective memory) and recognition (objective memory) of the meaning of the words that had been used as primes in the preceding pLDT, such that the integration analyses could be applied only to those words whose meaning had been both learned and retained. At the end of the experiment on Day 2, participants completed a questionnaire about learning strategies, learning preference, and number of hours slept between the two sessions. Furthermore, participants were asked how tired they were on a scale of 1–5 just prior to each pLDT.

### Materials

#### Study words and their definitions

Study words consisted of 128 Dutch concrete nouns, all lemmas with one possible spelling. Half of these were very uncommon words (frequency < 1 per million), and the other half were commonly known but low-frequency words (*M*_frequency_ = 12.52 per million, frequency range = 1–43 per million). These final stimuli had been selected from two larger pools of Dutch words from a word database (CELEX [21], 167 uncommon words/150 common words), which were each judged by 11 independent raters not involved in the experiment. We selected 64 words whose form and meaning were unknown to all the raters and assigned them to the “novel-word” condition. The form and meaning of the 64 words chosen for the “known-word” condition were known by all raters.

Definitions of the study words were taken from a commonly used Dutch dictionary [22] containing over 280.000 head words at the time of use. Some definitions were shortened by deleting unnecessary and/or complicated words to keep definition length similar over conditions. Spoken versions of the definitions were digitally recorded by a young female native Dutch speaker and edited with PRAAT [23]. Tempo was adjusted with preservation of pitch using Audacity [24]. Volume was equalized with PRAAT. During study, participants were allowed to alter the volume to their preference.

#### Targets for the primed lexical decision task (pLDT)

Both the novel and the known study words (64 each) were divided into two lists of 32 words each to serve as primes for the pLDT. The mean number of letters, syllables, orthographic Levenshtein distance 20 (OLD20, a measure of orthographic neighborhood density), and number of words in the definitions did not significantly differ between lists (S1 Table). For each prime word, there were three types of target words: one semantically related target, one semantically unrelated target, and two pseudoword targets (examples in Table 1). This resulted in 128 prime-target combinations per list. Note that none of the targets occurred during study (neither as study words nor as a part of the definitions) in order to avoid the possibility that RTs for the lexical decision would be influenced by word repetition, and to prevent episodic rather than semantic priming.

**Table 1:** Example for Each Type of Word Pair Used in the primed Lexical Decision Task.

	Related target	Unrelated target	Pseudoword target 1	Pseudoword target 2
Novel prime	sambar-gewei	sambar-hengel	sambar-gelie	sambar-reugel
	*sambar-antlers*	*sambar-fishing rod*	*sambar-gelie*	*sambar-reugel*
Known prime	vulkaan-lava	vulkaan-getal	vulkaan-dama	vulkaan-gepat
	*volcano-lava*	*volcano-number*	*volcano-dama*	*volcano-gepat*

*Note*. Italic words are English translations of Dutch words. Pseudoword targets do not exist as real words in the Dutch language and they were not pseudohomophones.

To create one semantically related target for each prime word, the previously mentioned independent raters were asked to give the first five conceptually related words that came to mind when reading the study/prime words with their definitions. This was done after they had judged these words on knowledge of meaning. This method of target generation could result in stronger prime-target relationships for the known compared to the novel primes, but should not affect the interpretation of the data since our interest was a comparison within the novel condition, and not between the novel and known condition. The selected target words were generated by at least two raters, but frequency of occurrence as listed in CELEX was not high (S2 Table). We selected directly related targets (volcano-lava) and avoided indirect associations (mountain-(volcano)-lava). Types of semantic relations used were synonyms, almost-synonyms (perceptual or functional), superordinates (mammal-dog), subordinates (dog-mammal), coordinates (dog-cat), functionally related (broom-sweep), instrumentally related (broom-floor), perceptual property (canary-yellow), spatial property (canary-cage), holomony (forest-tree), and meronymy (tree-forest). Some prime-target pairs shared multiple semantic relations. Types of semantic relations were equally distributed among lists.

To create the semantically unrelated word pairs, prime and target words were recombined such that they did not share a semantic relation. First, the targets were pseudorandomized over primes and recombined when necessary (face validity). Next, we confirmed that none of the unrelated target words were generated by any of the independent raters when they were asked to come up with semantically associated words. In none of the lists did the mean Levenshtein Distance (S3 Table), a measure of (orthographic) similarity between two words, differ between related and unrelated prime-target combinations (Mann-Whitney tests, all *p* ≥ .390).

Pseudowords served as non-word fillers for the lexical decision task. These ensured participants would make a semantic decision on every target (existing/non-existing). From each target word, we generated two pseudowords that obeyed Dutch phonotactic constraints using the program “Wuggy” [25]. This program allows for optimal pseudoword generation, taking into account subsyllabic structure and transition frequencies between subsyllabic elements (onset, nucleus, and coda). Word length (letters + syllables, S2 Table) and length of subsyllabic segments was kept identical between each existing target and its two matched pseudowords.

### Tasks

Assignment of left and right buttons to positive (known/existing) and negative (unknown/not existing) response options was kept constant for each participant throughout the tasks, and was counterbalanced across participants. In all tasks, participants had to respond via a keyboard, except in the pLDT for which a button box was used.

#### Knowledge of meaning test

The study words (64 novel, 64 known) were shown one by one on a computer monitor in random order, and the participant had to indicate whether he or she knew the meaning of the word or not by pressing a button corresponding to either a “known” or a “unknown” response. If novel words were responded to as “known” or if known words were responded to as “unknown”, the participant was asked what the word meant to confirm whether the given response was appropriate or not.

#### Study

In two rounds, participants studied both novel and known Dutch written words with their definitions (word meaning). The definitions were either visually or aurally presented, depending on the modality group participants were assigned to. Presentation time of a written definition was equal to its spoken counterpart. The rationale for presenting only word meaning in different modalities (written or spoken) and not word form is that we used visual presentation of the word forms in the pLDTs. In this way, both modality groups were exposed to the written word forms at study. Learning was largely self-paced: participants could decide when they wanted to see/hear a definition, but presentation length of the definition was fixed.

To make sure that all words and their definitions were encountered, in round 1, all 128 study words were presented once each, in random order. When a word was shown on the screen, the participant could press a button to see the definition of the word on the computer screen (written group) or hear it via loudspeakers (spoken group; meanwhile a blank screen was presented). Then the next word appeared on the screen.

In round 2, words were again presented serially in random order. For each word, the participant pressed the key to see/hear the definition, and after the definition presentation, three choices were shown: 1) See/hear the definition again (this option can be repeated as often as wanted); 2) Go on to the next word (the current word will appear again in the next cycle); 3) Remove the word from the to-be-learned list (the word will not appear in the following cycles). All words and definitions were encountered at least twice, once in round 1, and once in round 2 before they were omitted from the learning list. Participants were instructed to learn the meaning of all words, and only choose option 3 if they knew the meaning and thought they would not forget it. Participants were also told that they did not have to know the exact wording of the definitions. For every new cycle, the words that remained in the list were presented in random order. In round 2, participants could press a pause button whenever they wanted to take a break. The total study phase lasted until all words were omitted from the learning list or until a maximum of 2 hours (including breaks) was reached. After the study phase, participants were obliged to take a small break (5–10 min).

#### Primed Lexical Decision Task (pLDT)

For the pLDT on Day 1, one novel-prime/target list (N1 or N2) and one known-prime/target list (K1 or K2) were combined. This resulted in 256 word pairs of which the order was pseudo-randomized, such that the same prime did not occur in the same quarter of the experiment, and the same target (once used in a related and once in an unrelated prime-target combination) did not occur in the same half of the LDT. In each quarter, an equal number of prime-target relation types (related, unrelated, pseudoword (x2)) was shown in a randomized order. The two remaining lists (combined in an equal manner) were presented in the pLDT on Day 2. Combinations of lists ([Day 1/Day 2]: [N1+K1/N2+K2], [N2+K2/N1+K1], [N1+K2/N2+K1], [N2+K1/N1+K2]) were counterbalanced across participants, such that each prime-target combination was shown an equal number of times on Day 1 as on Day 2. Both the word pair pseudo-randomization and the list combinations were identical for the two modality groups.

A trial (see Fig 1 for an example) started with presentation of a fixation star (800 ms), followed by the prime word (250 ms) and then the target word (1000 ms). Participants were instructed to judge whether the second string of letters was an existing word or not (lexical decision) and to respond as fast and accurate as possible. Answers given outside the target presentation window were categorized as missed. An inter-trial interval of 2500 ms was included to minimize the possibility of cross-trial priming. RTs were measured from target onset until the button press. Before every pLDT, a practice pLDT of 20 trials was conducted with words that were not used in the actual pLDT, and with the response windows reduced to 800 ms to promote fast responses during the actual experiment.

#### Meaning recall test

Studied words that appeared in the preceding pLDT (32 novel, 32 known) were shown one by one on a screen in random order, and the participant had to indicate whether or not he or she knew the meaning of the word by pressing a button corresponding to either a “known” or an “unknown” response.

#### Meaning recognition test

Studied words that appeared in the preceding pLDT were presented one at a time. For each word, there were four definition options that appeared upon a corresponding button press. These were shortened versions of the study word definitions. Participants were allowed to press the corresponding buttons consecutively, for as many times as needed, before choosing the definition that they thought belonged to the presented word. Answers were given by pressing one of the four answer buttons and were followed by the next trial. To keep study and test modality equal, the shortened definitions were presented in written format for the written group, and in spoken format for the spoken group. The presentation time for a shortened written definition was matched with its spoken version. At the end, participants were shown the amount of correct answers.

### Analyses of Semantic Integration

Semantic integration was assessed by measuring the magnitude of the priming effects observed in the pLDTs. To ensure that all words in the novel condition were novel before but acquired during study, and to ensure that the known-condition words were actually known, trials were excluded from further analyses if the prime was incorrectly judged at the knowledge of meaning task, the meaning recall task, or the meaning recognition task. PLDT trials were also excluded if an existing target word was incorrectly judged as being a pseudoword. For each participant, RTs exceeding the range [*M*_condition_ **+/-** 3.29 *SD*] were excluded as outliers, because these scores are not expected to occur (probability of 0.001 in a normal distribution) [26]. Applying the Van Selst and Jolicoeur [27] procedure for outlier detection (non-recursive with shifting z-criterion) did not change the results in any substantial way. If a *p*_*VS&J*_-value crossed the significance boundary as compared to the original *p*-value, this has been indicated. To calculate participant-specific priming effects, the mean RT for the related prime-target combinations was subtracted from the mean RT for the unrelated prime-target combinations. To determine mean priming effects on the group level, the means of the participant-specific priming effects were computed. Participants were excluded from the group analyses if they were left with fewer than five related and/or unrelated trial-RTs for calculating the priming effect in at least one of the conditions (*M*_amount of trial-RTs per condition that entered the analyses_ (*SD*): related: 26 (3); unrelated: 25 (4); max = 32), or if a (difference between relevant) priming effect(s) exceeded the range [*M*_(difference between) condition(s)_ **+/-** 3.29 *SD*]. The first criterion resulted in two participants from the written group being excluded. The second criterion did not generate additional removals. For the by-items analyses an analogous procedure was used, resulting in exclusion of 14 items in the novel and two items in the known condition, because there were fewer than five related and/or unrelated participant-RTs left for calculating at least one of the different priming effects (*M*_amount of participant-RTs per condition that entered the analyses_ (*SD*): related: 13 (3); unrelated: 13 (3); max = 16). Error-priming effects were based on proportion incorrect, after removal of all misses and trials for which the prime was incorrectly judged at familiarity, recall, or recognition (included correct: 78% of total data; included incorrect: 5%; excluded: 17%; error-priming effect = (proportion incorrect for unrelated prime-target combinations)–(proportion incorrect for related prime-target combinations)).

## Results

### Study and Control Measurements

The following means are based on the 62 participants that were included in the semantic integration analyses (Learning System 2). Including the two excluded participants did not change the pattern of findings. The study phase (round 1 + round 2, excluding instructions and pauses) was on average completed in 1 hr 45 min (*SD* = 17 min) (*M*_study time_ in hr:min (*SD*): written: 1:43 (0:17); spoken: 1:46 (0:18)). Sixteen participants in the written group and 13 participants in the spoken group made full use of the allotted study time (i.e., 2 hrs). On average a word’s definition was presented 5.25 times (*SD* = 1.07) during study (*M* (*SD*): novel written: 7.97 (2.23); novel spoken: 8.20 (2.17); known written: 2.44 (0.59); known spoken: 2.40 (0.67)). The mean duration of a presented definition was 4.21 s (0.16) (*M* (*SD*) in s: novel written: 4.06 (0.16); novel spoken: 4.08 (0.17); known written: 4.68 (0.09); known spoken: 4.66 (0.08)). Study length, mean number of presentations per definition and mean duration of a presented definition did not differ between modality groups (Mann-Whitney tests, all *p* ≥ .185). Neither did the modality groups differ on the control measurements: learning style preference (*five point Likert item*: *1 = high preference for learning in written language*, *5 = high preference for spoken*; *M* (*SD*): written: 2.33 (1.03); spoken: 2.22 (0.91); *p* = .747), fatigue (*five point Likert item*: *1 = mentally completely tired*, *5 = mentally completely fresh*; *M* (*SD*): written Day 1: 3.17 (0.75); spoken Day 1: 2.97 (0.78); written Day 2: 3.73 (0.87); spoken Day 2: 3.28 (0.99); Day 1: *p* = .282, Day 2: *p* = .057, Day 2–Day 1: *p* = .389), and number of hours slept between the two sessions (*M* (*SD*): written (*n* = 23 due to a lack of sleep log data in 7 participants): 7.43 (1.01); spoken (*n* = 32): 7.82 (1.19); *p* = .184) (all Mann-Whitney tests). In short, the written and spoken study modality group showed similar study characteristics and did not differ on the control measurements.

### Acquisition (Learning System 1)

Table 2 shows the knowledge of studied word meanings at several time points. As expected, participants were unfamiliar with the meaning of novel words before study (0.6% known), whereas the meaning of known words were known (99.9%). Shortly after study, participants’ subjective retrieval of the definition was 75.4% for the novel words and 99.9% for the known words. On Day 2, subjective retrieval of novel word meanings decreased to 66.4%, whereas retrieval of known word meanings remained at 99.9%. Objective recognition (as tested with a 4-alternative forced-choice meaning recognition test) of novel as well as known word meanings was at ceiling level (> 95%) on both days. The recall and recognition scores for novel and known words on both Day 1 and Day 2 did not differ between the written and spoken condition (Mann-Whitney tests, all *p* ≥ .282).

**Table 2:** Mean Percentage (*SD*) of Word Meanings Responded as Known.

		Before study	After pLDT		After pLDT	
		Day 1	Day 1		Day 2	
Study modality	Prime	Knowledge of meaning	Meaning recall	Meaning recognition	Meaning recall	Meaning recognition
Written	Novel	0.9 (1.7)	76.1 (21.7)	97.8 (3.6)	68.6 (24.3)	95.8 (6.7)
(n = 32)	Known	99.9 (0.4)	99.9 (0.6)	99.8 (0.8)	99.9 (0.6)	99.7 (0.9)
Spoken	Novel	0.3 (0.8)	74.7 (19.2)	97.2 (5.2)	64.3 (19.9)	96.3 (5.2)
(n = 32)	Known	99.9 (0.5)	99.9 (0.6)	98.7 (4.6)	99.9 (0.6)	99.7 (0.9)
Combined	Novel	0.6 (1.3)	75.4 (20.3)	97.5 (4.4)	66.4 (22.1)	96.0 (6.0)
(N = 64)	Known	99.9 (0.4)	99.9 (0.5)	99.3 (3.3)	99.9 (0.5)	99.7 (0.9)

A mixed within-between participants analysis of variance (ANOVA) on subjective recall rate with Day (1/2) as within-participants factor and Modality (written/spoken) as between-participants factor, showed that there was no main effect of Modality (*p* = .585) on recall of the novel word meanings. Recall rates were significantly lower on Day 2 (main effect of Day: *F*(1,62) = 41.26, *p* < .001), but the amount of forgetting (*M*_difference Day1-Day2_ (*SD*) = 9.0% (11.2%)) did not differ between modalities (interaction between Day and Modality: *p* = .299). Correlation analyses using Spearman’s r showed that age did not significantly correlate with learning performance (recall: all *p* ≥ .284; recognition: all *p* ≥ .120).

In short, novel word meanings were not yet known before, but largely acquired during the study session and not forgotten much on Day 2, in either modality. Word meanings of the known words were already known before study and were not forgotten. None of the above conclusions changed if we excluded the two participants that had to be excluded for the analyses on the pLDT.

### Semantic Integration (Learning System 2)

Table 3 shows the mean priming effects, and the overall mean RTs for the pLDT. Performance in the pLDT was at ceiling level (92.3% correct, 6.2% incorrect, 1.6% missed).

**Table 3:** Mean Reaction Times (*SD*) and Mean Priming Effects (*SD*) in the primed Lexical Decision Task.

			Day 1		Day 2	
Modality group	Prime	Prime-target relation	RT	Priming effect^a^	RT	Priming effect
Written	Novel	Related	590 (56)	**5 (35)**	555 (51)	**17 (23)**
(n = 30)		Unrelated	594 (59)		572 (55)	
	Known	Related	545 (59)	31 (21)	528 (54)	27 (21)
		Unrelated	576 (57)		555 (56)	
Spoken	Novel	Related	595 (56)	**-5 (32)**	570 (52)	**3 (27)**
(n = 32)		Unrelated	590 (54)		573 (62)	
	Known	Related	554 (46)	21 (34)	534 (57)	26 (28)
		Unrelated	575 (44)		560 (51)	
Combined	Novel	Related	592 (56)	**0 (33)**	563 (51)	**10 (26)**
(N = 64)		Unrelated	592 (56)		573 (58)	
	Known	Related	550 (53)	26 (28)	531 (55)	26 (25)
		Unrelated	576 (50)		557 (53)	

*Note*. *SD* = Standard Deviation; Both reaction times and priming effects are presented in ms.

^a^Priming effect does not always match “*M*_RT unrelated_—*M*_RT related_” because the difference was calculated for each participant before averaging.

**Fig 2:**
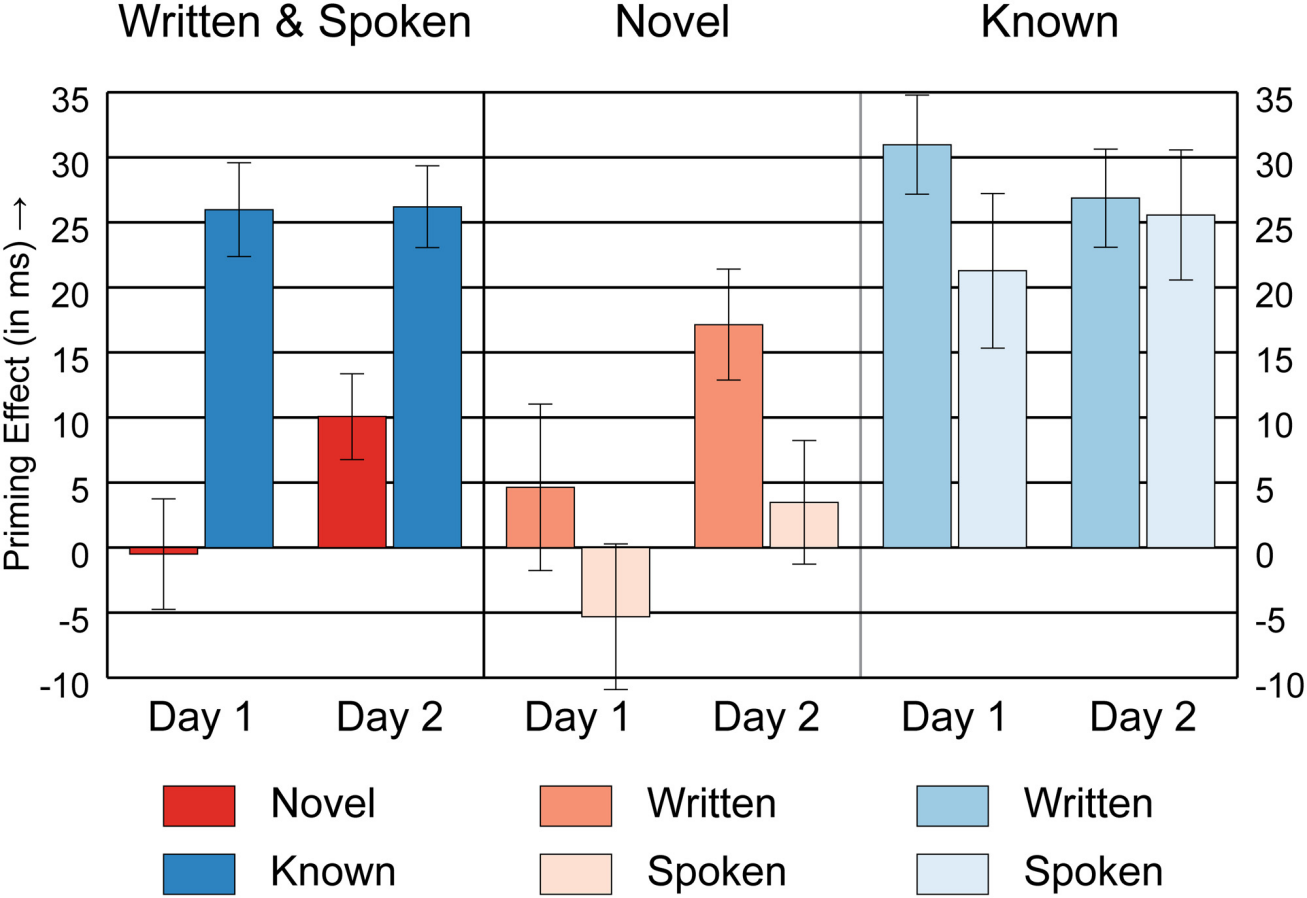
Priming Effects. Mean priming effects (± standard error of the mean) for: novel and known primes (left), novel primes separately for written and spoken study modality (middle), and known primes separately for the two modalities (right). Participant-specific priming effects were calculated by subtracting the mean RT for semantically related from the mean RT for semantically unrelated targets.

Novel words were expected to become integrated into the semantic lexicon over time and therefore to show an increase in priming over days. A mixed ANOVA (Day x Modality) showed a main effect of Day; for newly learned words, the priming effect increased over time (Fig 2 left, red bars; Day 1: *M* = 0 ms, Day 2: *M* = 10 ms; *F*(1,60) = 4.13, *p* = .047, η_p_^2^ = .064), suggesting that integration took place between the two sessions. The integration process did not significantly differ between study modalities, as we found no interaction between Day and Modality (Fig 2 middle; *p* = .724). There was, however, a main effect of Modality; in general, priming effects were larger for the written modality compared to the spoken modality (Fig 2 middle; *F*(1,60) = 4.80, *p* = .032, η_p_^2^ = .074).

Following our hypothesis, we further investigated the priming effect separately for each day with one-sample t-tests. For both modalities, there was no priming effect on Day 1 (written: *p* = .475; spoken: *p* = .350; combined: *p* = .907), whereas on Day 2 the novel words showed a significant priming effect for the written condition (*t*(29) = 4.02, *p* < .001, Cohen’s *d* = 0.733), but not for the spoken condition (*p* = .470; combined: *t*(61) = 3.06, *p* = .003, Cohen’s *d* = 0.388). Direct comparisons between the two study modalities with independent t-tests showed that the amount of priming did not differ on Day 1 (*p* = .245), but was significantly larger for the written condition on Day 2 (*t*(60) = 2.13, *p* = .037, *p*_*VS&J*_ = .073, Cohen’s *d* = 0.542). The pattern of results did not change when including the non-remembered trials or the excluded participants. Correlation analyses using Spearman’s *r* showed that age did not significantly correlate with novel word performance in the pLDT ((difference between) priming effects; all *p* ≥ .407).

Known word meanings were expected to be already integrated. Accordingly, one-sample t-tests revealed significant priming effects for known words on both days for both modalities (Fig 2 right; written Day 1: *t*(29) = 8.12, *p* < .001, Cohen’s *d* = 1.483; spoken Day 1: *t*(31) = 3.58, *p* = .001, Cohen’s *d* = 0.633; combined Day 1: *t*(61) = 7.20, *p* < .001, Cohen’s *d* = 0.915; written Day 2: *t*(29) = 7.12, *p* < .001, Cohen’s *d* = 1.299; spoken Day 2: *t*(31) = 5.12, *p* < .001, Cohen’s *d* = 0.904; combined Day 2: *t*(61) = 8.35, *p* < .001, Cohen’s *d* = 1.061). Furthermore, a mixed ANOVA (Day x Modality) on priming effects of the known condition showed no significant main effect for Day (Fig 2 left, blue bars; *p* = .985), Modality (Fig 2 right; *p* = .253), or an interaction between the two (Fig 2 right; *p* = .386). This suggests that the Day 1/Day 2 increase in priming effect for novel primes cannot be explained by task repetition (practice effects), and that the modality effects observed for novel primes cannot be explained by a higher basic level of priming effects in the written modality group.

By-items analyses showed that on the item level, for the novel words, the main effect of both Day and Modality were no longer significant (Day: *p* = .122; Modality: *p* = .223; Day x Modality: *p* = .636), but showed the same numerical pattern as the by-participants priming effects (novel; written Day 1: *M* = 2 ms; spoken Day 1: *M* = -2 ms; combined Day 1: *M* = 0 ms; written Day 2: *M* = 14 ms; spoken Day 2: *M* = 4 ms; combined Day 2: *M* = 9 ms). One-sample t-tests confirmed the effects found in the by-participants analyses (novel: written Day 1: *p* = .723; spoken Day 1: *p* = .770; combined Day 1: *p* = .979; written Day 2: *t*(49) = 2.60, *p* = .012, Cohen’s *d* = 0.368; spoken Day 2: *p* = .432; combined Day 2: *t*(99) = 2.37, *p* = .020, *p*_*VS&J*_ = .061, Cohen’s *d* = 0.238). For the known words, the by-item analyses completely followed the by-participants analyses, with no main or interaction effects (Day: *p* = .556; Modality: *p* = .340; Day x Modality: *p* = .334), the same pattern of priming effects (known; written Day 1: *M* = 31 ms; spoken Day 1: *M* = 23 ms; combined Day 1: *M* = 27 ms; written Day 2: *M* = 30 ms; spoken Day 2: *M* = 30 ms; combined Day 2: *M* = 30 ms) and priming for all conditions (known: written Day 1: *t*(61) = 6.85, *p* < .001, Cohen’s *d* = 0.870; spoken Day 1: *t*(61) = 4.28, *p* < .001, Cohen’s *d* = 0.543; combined Day 1: *t*(123) = 7.69, *p* < .001, Cohen’s *d* = 0.690; written Day 2: *t*(61) = 6.03, *p* < .001, Cohen’s *d* = 0.765; spoken Day 2: *t*(61) = 5.69, *p* < .001, Cohen’s *d* = 0.722; combined Day 2: *t*(123) = 8.30, *p* < .001, Cohen’s *d* = 0.745).

The accuracy data of the above included participants partly confirmed the RT analyses. A lower proportion of errors could be expected for semantically related word pairs, reflected as a positive error-priming effect. For the novel words, a mixed ANOVA (Day x Modality) on the error-priming effects, showed a significant increase over days (*F*(1,60) = 5.98, *p* = .017, η_p_^2^ = .091), no main effect of modality (*p* = .901), and no interaction between Day and Modality (*p* = .111) (error-priming effects in proportion of errors; novel: written Day 1: *M* = .01; spoken Day 1: *M* = -.01; combined Day 1: *M* = -.00; written Day 2: *M* = .02; spoken Day 2: *M* = .04; combined Day 2: *M* = .03). For the known words, there were no significant effects for Day (*p* = .084), Modality (*p* = .919), and Day x Modality (*p* = .080) (error-priming effects in proportion of errors; known: written Day 1: *M* = .04; spoken Day 1: *M* = .02; combined Day 1: *M* = .03; written Day 2: *M* = .06; spoken Day 2: *M* = .04; combined Day 2: *M* = .05).

## Discussion

The two goals of the present study were to investigate whether newly acquired words require time to become integrated into the semantic network, and to reveal possible effects of study modality on this integration process. We observed a significant increase in semantic priming over time: newly learned words did not prime semantically related words directly after study, but did so after a delay of approximately 24 hours. This finding indicates that novel words are indeed semantically integrated via a slow learning process that follows rapid episodic acquisition. Although there was no significant difference in the strength of this time-dependent priming increase between modality groups, the effect appears to be driven by the written study group. We observed a positive priming effect for words studied with a written definition after 24 hours, which was not the case for words studied with a spoken definition. Correspondingly, a main effect of study modality reflected larger overall priming effects for the written group.

### Integration Over Time

Davis and Gaskell [2] suggested that word learning occurs via two complementary learning systems (CLS): 1) rapid acquisition via the medial temporal lobe and 2) slower integration supported by the neocortex. Abundant empirical findings have already shown that integration of word *forms* into the mental lexicon, measured in terms of lexical competition, benefits from a consolidation period that includes sleep [7] although under some learning conditions sleep might not be required [28]. Integration of word meanings, on the other hand, is relatively understudied [11, 14]. Our results provide further evidence for the CLS model by showing that not only word *forms* but also word *meanings* are slowly integrated. Put differently, integration of words into the neocortical *semantic* lexicon requires time.

Already after 24 hours, we observed priming effects from newly learned words to related existing words, whereas signs of semantic integration were observed after 7 days in the studies of Tamminen and Gaskell [11] and Clay et al. [14]. Tamminen and Gaskell—whose study is most comparable to ours—did not find priming to be influenced by the duration of the consolidation period (0, 1 or 7 days). This could have been the result of a lack of statistical power, because when the priming effect was tested with a combination of trials from two experiments, results pointed towards more integration after a 7-day versus a 0-day consolidation period (the 1-day versus 0-day consolidation period was not tested). We, on the other hand, observed that the newly learned words influenced the processing of related target words after 24 hrs. The difference in length of the consolidation period that seems to be necessary for semantic integration to occur can possibly be explained by the relevance of the study materials. Relevance of the studied information has an impact on the consolidation process [29, 30]. In our study, participants were explicitly told that they would be tested on the word meanings. On top of that, and contrary to the other two studies, the study words were existing words that participants could use outside of the laboratory, which likely increased relevance and therefore incorporation [31]. In short, both the instruction and the use of meaningful existing words may have promoted rapid integration by increasing the relevance of the new memories.

Our findings are of interest from the broader perspective of memory consolidation. Historically, consolidation was used to define the process in which an initially labile memory trace is *stabilized*. In other words, the original memory trace is strengthened such that it becomes less prone to interference and decay. In accordance with a more recent view on the function of consolidation [3, 4, 32–34], our data show that a memory trace can also change qualitatively with time: newly learned words influenced processing of familiar words only after a delay, indicating that semantic associations between newly learned and existing words are formed during a consolidation period. For the integration measurements, on both Day 1 and Day 2, we only considered words whose meanings were accessible as tested in the recall/recognition tests. Knowledge of a word’s meaning was not sufficient for causing semantic priming, as we observed no significant priming on Day 1. However, with time, the status of the newly learned words changed such that they started to influence the processing of semantic neighbor words, suggesting that qualitative changes occurred between the novel words and semantically related words. The increased priming effect for novel words on Day 2 cannot be explained by task repetition, because no increase was observed in the known condition (i.e., for the words that already had stable semantic representations before the experiment).

Overall, our data suggest that the newly formed memory traces undergo qualitative changes during consolidation, which is in accordance with both the CLS account of word learning and a more recent view on the function of consolidation [3, 4]. In the following, limitations and future studies are discussed.

By-items analyses showed weaker effects compared to by-participants analyses. Therefore, we need to be cautious in generalizing our results across a larger population of words. The weaker effects in the by-items analyses can be explained by the fact that in semantic priming experiments the between-participants variability in RTs is relatively large as compared to the effect of interest, namely the semantic priming effect which is measured as “unrelated RT”–“related RT” (within-participants difference score). Because a by-items analysis does not control for the inter-participant reaction time differences, the probability of finding significant effects is reduced. We both matched item variability across the prime/target lists and counterbalanced these lists among participants, such that we could interpret our data based on the by-participants analyses alone [35]. That being said, generalizing our results to a larger sample of words should be done with caution.

We aimed for capturing the automatic spreading of activity across semantic memory by using a short stimulus onset asynchrony of 250 ms between prime and target [36]. However, we cannot fully discard that strategic processing contributed to the priming effect. If one wants to focus on automatic processing, a possibility is to use masked priming, as was done in the second experiment of Tamminen and Gaskel [11].

The consolidation opportunity in our study contained a period of sleep, but with our design, we cannot dissociate the effects of sleep from time. In order to dissociate sleep from time, future research could for instance test a sleep versus a no-sleep group.

### Study Modality Effects on Integration

We found a significant main effect of study modality on integration: priming effects were largest when word meanings had been studied in written format. Furthermore, the priming effect on Day 2 was significant for the written, but not for the spoken study modality group, with the difference in priming effects between the two modalities being significant. The latter finding suggests that the integration process particularly benefits material acquired in the written modality, as was expected. This interpretation, however, has to be taken with caution as we did not find a significant interaction between Modality and Day, which seems to indicate that the priming effect increase over time was stable across the two groups. Nonetheless, overall semantic integration was greater for written information.

Why did the written study modality condition show larger priming effects than the spoken condition? Because written words are less obtrusive in nature than spoken words, the written condition could lead to greater availability of both time and memory resources for rehearsal or reminiscence during the study period. This may result in a boost in integration. During study, participants in both modality groups reported to make use of the retrieval method [37, 38]: when they saw the word on the screen, they mentally tried to retrieve its meaning and then checked their accuracy by seeing/hearing the correct definition. If the mentally retrieved meaning was the correct one, the participant presumably realized this at a relatively early stage of the definition presentation. Intuitively, it seems to be easier to not look at the remainder of the written definition than to ignore the ongoing spoken definition. As such, the written group had more freedom to devote their time to mental rehearsal or reminiscence, leading to enhanced integration. One could argue that verbal working memory was overtaxed in the spoken condition. If so, one would assume that the definitions, especially the longer ones, were more often repeated during study in the spoken condition. This, however, was not the case. Immediate memory performance did not differ between modality groups, further strengthening the idea that verbal working memory was not overburdened for the spoken modality group during study of the novel words.

Written words are also less fleeting in nature than spoken words, which could lead to more focused encoding. A certain depth or richness of episodic encoding may be necessary for a memory trace to become a candidate for semantic integration. Research using eye movement recording has shown that participants focus on the most informative parts of a text when they read [39]. Even though the number of definition exposures was equal for the written and spoken condition, with presentation length of written definitions matched to spoken definitions, participants had the chance to focus on the relevant part of the definition in the written condition because all information was simultaneously present, whereas this was not possible for the spoken condition. In other words, a participant in the written condition had more control over where to attend, resulting in a more focused episodic encoding for the written condition. The similarity of the recall and recognition data argue against this “more focused encoding” interpretation, although it is possible that our acquisition measurements were not sensitive enough to pick up differences in encoding strength between the modality groups.

Clarifying the effects of study modality on vocabulary acquisition and integration is of practical relevance (see [40], for word form consolidation across modalities). If written word meanings are faster or more tightly integrated into the semantic network, then educators could include more written study material in their lessons. We mention two possible explanations for the observed benefit in integration for written study material above, but further investigation is necessary to elucidate the exact nature of the study modality effects. One may, for example, investigate the time course of integration by including more measurement points to disentangle whether integration for written material is stronger or starts earlier. Stronger integration of written material would predict a difference in priming effects between the two modality groups even at longer delays, whereas earlier integration would predict the modality difference to decrease or disappear after a longer consolidation interval. Note furthermore that, contrary to other studies [18, 20], we kept study and test modality congruent for both the written and spoken condition, by only manipulating study modality of the word *meanings* (written/spoken), not of the word *forms*. We used the written modality for the crucial test, and for this reason both groups were exposed to written word forms during study. For a more complete understanding of the modality effect, it is necessary to also investigate spoken word forms as the test modality. Another interesting avenue of research is to investigate whether the modality effect is reduced (or would even be reversed) in children, who have less expertise in reading, and might therefore make less use of the benefits that written language offers.

## Conclusion

Our data provide evidence for the view that integration of novel words into the semantic lexicon occurs over a time period that extends past the study phase itself. This adds to recent literature on vocabulary learning, by showing that not only integration of word form, but also integration of word meaning requires time. From a broader perspective, our data support the notion that memory traces change qualitatively with consolidation, and that integration of new information into an existing corpus of knowledge is part of this consolidation process. Furthermore, there appears to be an advantage for written input over spoken input in terms of semantic integration. After initial acquisition, learning is not yet finished. A memory trace evolves and its evolution seems to depend on the modality in which it is encoded.

## Supporting Information

S1 Dataset Dataset. (ZIP)

S1 Table Statistics on Words Used as Primes in the primed Lexical Decision Task (*n* = 32 per list). (DOCX)

S2 Table Statistics on Words Used as Targets in the primed Lexical Decision Task (*n* = 128 per list: 32 related, 32 unrelated and 64 pseudoword targets). (DOCX)

S3 Table Mean Levenshtein Distance Between Prime and Target in the primed Lexical Decision Task. (DOCX)
