# Supplementary material for: Learning Word Meanings: Overnight Integration and Study Modality Effects
Source: PLoS One. 2015 May 19;10(5):e0124926. doi: 10.1371/journal.pone.0124926 (PMC4437978; doi:10.1371/journal.pone.0124926)
Supplement: S1 Table — (DOCX) [file pone.0124926.s002.docx]

**S1 Table. Statistics on Words Used as Primes in the primed Lexical Decision Task (*n* = 32 per list).**

| List^a^ | *M* and *SD* | Letters | Syllables | Frequency | OLD20 | *n* Words definition | *versus* List^a^ | *U* and *p* | Letters | Syllables | Frequency | OLD20 | *n* Words definition |
| --- | --- | --- | --- | --- | --- | --- | --- | --- | --- | --- | --- | --- | --- |
| **N1** | ***M*** | **5.31** | **1.63** | **0.00^b^** | **1.75** | **9.22** | N2 | *U* | 532.50 | 567.00 | 512.00 | 551.00 | 535.00 |
|  | ***SD*** | **1.84** | **0.98** | **0.00** | **0.74** | **6.18** |  | *p* | .778 | .392 | 1.000 | .599 | .75 |
|  |  |  |  |  |  |  | K1 | *U* | 561.00 | 558.50 | 1,024.00 | 476.00 | 653.00 |
|  |  |  |  |  |  |  |  | *p* | .500 | .478 | <.001* | .628 | .058 |
|  |  |  |  |  |  |  | K2 | *U* | 538.50 | 538.00 | 1,024.00 | 483.50 | 615.50 |
|  |  |  |  |  |  |  |  | *p* | .716 | .689 | <.001* | .701 | .164 |
| **N2** | ***M*** | **5.53** | **1.81** | **0.00^b^** | **1.82** | **9.75** | K1 | *U* | 524.50 | 499.50 | 1,024.00 | 445.50 | 624.50 |
|  | ***SD*** | **2.00** | **1.06** | **0.00** | **0.76** | **6.57** |  | *p* | .862 | .854 | <.001* | .371 | .130 |
|  |  |  |  |  |  |  | K2 | *U* | 508.00 | 477.00 | 1,024.00 | 464.00 | 587.00 |
|  |  |  |  |  |  |  |  | *p* | .956 | .603 | <.001* | .518 | .313 |
| **K1** | ***M*** | **5.34** | **1.66** | **12.28** | **1.60** | **10.97** | K2 | *U* | 478.00 | 482.50 | 498.50 | 519.50 | 476.50 |
|  | ***SD*** | **1.21** | **0.75** | **9.88** | **0.53** | **4.07** |  | *p* | .638 | .661 | .856 | .920 | .632 |
| **K2** | ***M*** | **5.22** | **1.56** | **12.75** | **1.58** | **10.78** |  |  |  |  |  |  |  |
|  | ***SD*** | **1.24** | **0.67** | **11.07** | **0.48** | **5.66** |  |  |  |  |  |  |  |

*Note.* *M* = Mean; *SD* = Standard Deviation; OLD20 = Orthographic Levenshtein Distance 20; *n* Words definition = number of words in the definition. *U* and *p* values are based on Mann-Whitney tests.

^a^N: novel-prime/target list; K: known-prime/target list. ^b^Occurrence < 1 per million.

* *p* < .05.
