# Supplementary material for: Learning Word Meanings: Overnight Integration and Study Modality Effects
Source: PLoS One. 2015 May 19;10(5):e0124926. doi: 10.1371/journal.pone.0124926 (PMC4437978; doi:10.1371/journal.pone.0124926)
Supplement: S2 Table — (DOCX) [file pone.0124926.s003.docx]

**S2 Table. Statistics on Words Used as Targets in the primed Lexical Decision Task (*n* = 128 per list: 32 related, 32 unrelated and 64 pseudoword targets).**

|  |  | Related, unrelated and pseudoword targets | | Related and unrelated targets^a^ | | | Pseudoword targets |  |  |  |  |  |  |  |
| --- | --- | --- | --- | --- | --- | --- | --- | --- | --- | --- | --- | --- | --- | --- |
| List^b^ | *M* and *SD* | Letters | Syllables | Frequency | Generated^c^ | OLD20 | OLD20^d^ | *versus* List^b^ | *U* and *p* | Letters | Syllables | Frequency | Generated | OLD20 (un)related targets |
| **N1** | ***M*** | **6.13** | **1.88** | **32.53** | **5.34** | **1.66** | **1.67** | N2 | *U* | 441.00 | 462.00 | 541.50 | 539.00 | 470.00 |
|  | ***SD*** | **1.93** | **0.71** | **61.61** | **1.82** | **0.51** | **0.59** |  | *p* | .332 | .465 | .691 | .713 | .572 |
|  |  |  |  |  |  |  |  | K1 | *U* | 405.50 | 434.00 | 478.50 | 620.50 | 401.50 |
|  |  |  |  |  |  |  |  |  | *p* | .147 | .259 | .652 | .139 | .136 |
|  |  |  |  |  |  |  |  | K2 | *U* | 469.50 | 402.00 | 439.50 | 507.50 | 458.00 |
|  |  |  |  |  |  |  |  |  | *p* | .562 | .109 | .329 | .951 | .467 |
| **N2** | ***M*** | **5.69** | **1.75** | **45.47** | **5.50** | **1.63** | **1.63** | K1 | *U* | 446.00 | 481.00 | 460.00 | 589.00 | 438.50 |
|  | ***SD*** | **1.64** | **0.72** | **69.50** | **2.03** | **0.60** | **0.66** |  | *p* | .366 | .652 | .484 | .294 | .322 |
|  |  |  |  |  |  |  |  | K2 | *U* | 549.00 | 451.00 | 434.00 | 477.00 | 499.50 |
|  |  |  |  |  |  |  |  |  | *p* | .611 | .370 | .293 | .634 | .867 |
| **K1** | ***M*** | **5.50** | **1.69** | **26.69** | **6.03** | **1.49** | **1.51** | K2 | *U* | 603.00 | 486.00 | 492.00 | 400.50 | 569.00 |
|  | ***SD*** | **2.18** | **0.78** | **54.61** | **1.80** | **0.51** | **0.57** |  | *p* | .214 | .701 | .787 | .130 | .442 |
| **K2** | ***M*** | **5.88** | **1.63** | **25.38** | **5.41** | **1.62** | **1.61** |  |  |  |  |  |  |  |
|  | ***SD*** | **1.68** | **0.79** | **59.77** | **2.21** | **0.64** | **0.64** |  |  |  |  |  |  |  |

*Note.* *M* = Mean; *SD* = Standard Deviation; OLD20 = Orthographic Levenshtein Distance 20. *U* and *p* values are based on Mann-Whitney tests.

^a^Targets in the related and unrelated conditions were identical. Only the semantic relation to the prime differed. ^b^N: novel-prime/target list; K: known-prime/target list. ^c^The amount of independent raters (*max* = 11) that produced the selected target word when asked for a conceptually related word to the prime. ^d^In none of the lists did OLD20 significantly differ between pseudoword targets and existing (related/unrelated) targets.
