# Supplementary material for: Learning Word Meanings: Overnight Integration and Study Modality Effects
Source: PLoS One. 2015 May 19;10(5):e0124926. doi: 10.1371/journal.pone.0124926 (PMC4437978; doi:10.1371/journal.pone.0124926)
Supplement: S3 Table — (DOCX) [file pone.0124926.s004.docx]

**S3 Table. Mean Levenshtein Distance Between Prime and Target in the primed Lexical Decision Task.**

|  |  | Prime-target relation | | |
| --- | --- | --- | --- | --- |
| List^a^ |  | Related | Unrelated | Pseudoword |
| N1 | *M* | 5.56 | 5.91 | 5.69 |
|  | *SD* | 1.63 | 1.67 | 1.60 |
| N2 | *M* | 6.09 | 5.78 | 5.80 |
|  | *SD* | 1.47 | 1.60 | 1.62 |
| K1 | *M* | 5.50 | 5.66 | 5.47 |
|  | *SD* | 1.76 | 1.49 | 1.59 |
| K2 | *M* | 5.38 | 5.75 | 5.56 |
|  | *SD* | 1.70 | 1.63 | 1.52 |

*Note.* *M* = Mean; *SD* = Standard Deviation.

^a^N: novel-prime/target list; K: known-prime/target list.
